# KLF5 inhibits angiogenesis in *PTEN*-deficient prostate cancer by attenuating AKT activation and subsequent HIF1α accumulation

**DOI:** 10.1186/s12943-015-0365-6

**Published:** 2015-04-21

**Authors:** Xinpei Ci, Changsheng Xing, Baotong Zhang, Zhiqian Zhang, Jenny Jianping Ni, Wei Zhou, Jin-Tang Dong

**Affiliations:** Department of Genetics and Cell Biology, College of Life Sciences, Nankai University, 94 Weijin Road, Tianjin, 300071 China; Department of Hematology and Medical Oncology, Emory Winship Cancer Institute, Emory University School of Medicine, 1365-C Clifton Road, Atlanta, GA 30322 USA

**Keywords:** KLF5, Angiogenesis, HIF1α, AKT, PTEN, Prostate cancer

## Abstract

**Background:**

KLF5 is a basic transcriptional factor that regulates multiple physiopathological processes. Our recent study showed that deletion of *Klf5* in mouse prostate promotes tumorigenesis initiated by the deletion of *Pten*. While molecular characterization of *Klf5*-null tumors suggested that angiogenesis was partially responsible for tumor promotion, the precise function and mechanism of *KLF5* deletion in prostate tumor angiogenesis remain unclear.

**Results:**

Applying histological staining to *Pten*-null mouse prostates, we observed that deletion of *Klf5* significantly increased the number of microvessels, accompanied by the upregulation of multiple angiogenesis-related genes based on microarray analysis with MetaCore software. In human umbilical vein endothelial cells (HuVECs), tube formation and migration, both of which are indicators of angiogenic activities, were decreased by conditioned media from PC-3 and DU 145 human prostate cancer cells with *KLF5* overexpression, but increased by media from cells with *KLF5* knockdown. HIF1α, a key angiogenesis inducer, was upregulated by KLF5 loss at the protein but not the mRNA level in both mouse tissues and human cell lines, as determined by immunohistochemical staining, real-time RT-PCR and Western blotting. Consistently, KLF5 loss also upregulated VEGF and PDGF, two pro-angiogenic mediators of HIF1α function, as analyzed by immunohistochemical staining in mouse tissues and ELISA in conditioned media. Mechanistically, AKT activity, which caused the accumulation of HIF1α, was increased by *KLF5* knockout or knockdown but decreased by *KLF5* overexpression. PI3K/AKT inhibitors consistently abolished the effects of *KLF5* knockdown on angiogenic activity, HIF1α accumulation, and VEGF and PDGF expression.

**Conclusion:**

KLF5 loss enhances tumor angiogenesis by attenuating PI3K/AKT signaling and subsequent accumulation of HIF1α in *PTEN* deficient prostate tumors.

**Electronic supplementary material:**

The online version of this article (doi:10.1186/s12943-015-0365-6) contains supplementary material, which is available to authorized users.

## Introduction

Angiogenesis, the process of forming new blood vessels from pre-existing vessels, is vital for development, tissue wound healing, and tumor initiation and progression [1]. Tumor angiogenesis is activated by multiple pro-angiogenic secretory factors including VEGF, PDGF-B, bFGF and TNF-α, which are all transcriptionally activated by hypoxia inducible factor 1 (HIF1) [2], composed of HIF1α and HIF1β subunits. HIF1α is mainly regulated at the protein level by the ubiquitin proteasome pathway, whose activation depends on the oxygen level, or by the activation of PI3K/AKT and MAPK signaling in a tumor, which is independent of oxygen level [2,3]. Targeting angiogenesis has been proposed as a therapeutic approach in prostate and other types of solid tumors for decades, and a number of candidate drugs have been tested in clinical trials. However, few of them have been officially approved for cancer treatment and their success has been limited [4]. A better understanding of the molecular basis of angiogenesis should improve the development of anti-angiogenesis-based cancer therapy.

*KLF5* (*Krüppel-like factor 5*, also known as *BTEB2*) encodes a basic transcription factor expressed in different tissues to regulate diverse cellular processes [5]. In tumorigenesis, KLF5 has been shown to play a context-dependent role. On one hand, its genetic locus is frequently deleted in human cancers [5,6]; a tumor suppressor function has been established in mouse models where *KLF5* expression suppresses tumor growth [7,8] and deletion of *Klf5* in mouse prostates promotes tumorigenesis initiated by the deletion of *Pten* [9]. On the other hand, interruption of KLF5 acetylation converts its function from that of a tumor suppressor to a tumor promoter, and multiple oncogenic pathways appear to be involved [8]. Recent studies also suggest that KLF5 could regulate angiogenesis. KLF5 is markedly induced in activated vascular smooth muscle cells and fibroblasts, deletion of *Klf5* in mice compromises vascular remodeling involving Pdgf-a [10], and loss of *Klf5* in mouse cornea epithelial cells results in abnormal neovascularization with elevated expression of angiogenesis-related genes [11,12]. In our recent study [9], we found that deletion of *Klf5* in *Pten*-null mouse prostates resulted in larger tumors without lumens and necrosis, which were present in tumors induced by *Pten* deletion alone [13] but with enhanced activation of AKT and ERK, which are reported to promote tumor angiogenesis [14,15]. It is thus possible that KLF5 modulates angiogenesis during prostatic tumorigenesis.

In this study, we tested whether and how KLF5 regulates angiogenesis in the context of *PTEN* loss in prostate cancer. We found that in *Pten*-null mouse prostate tumors, deletion of *Klf5* significantly promoted angiogenesis, and conditioned media from human prostate cancer cells with modulated *KLF5* expression affected tube formation and migration of human umbilical vein endothelial cells (HuVECs). Expression profiling and other analyses demonstrated that *Klf5* deletion led to the upregulation of multiple pro-angiogenic genes and accumulation of HIF1α, a transcription factor that stimulates angiogenesis. Additional experiments showed that accumulation of HIF1α induced by *KLF5* loss depended on the activation of the PI3K/AKT signaling pathway. These findings suggest that KLF5 loss promotes tumor angiogenesis by enhancing PI3K/AKT signaling and the subsequent accumulation of HIF1α in *PTEN* deficient prostate cancer.

## Results

### *Klf5* deletion promotes angiogenesis initiated by *Pten* deletion in mouse prostate tumors

**Figure 1:**
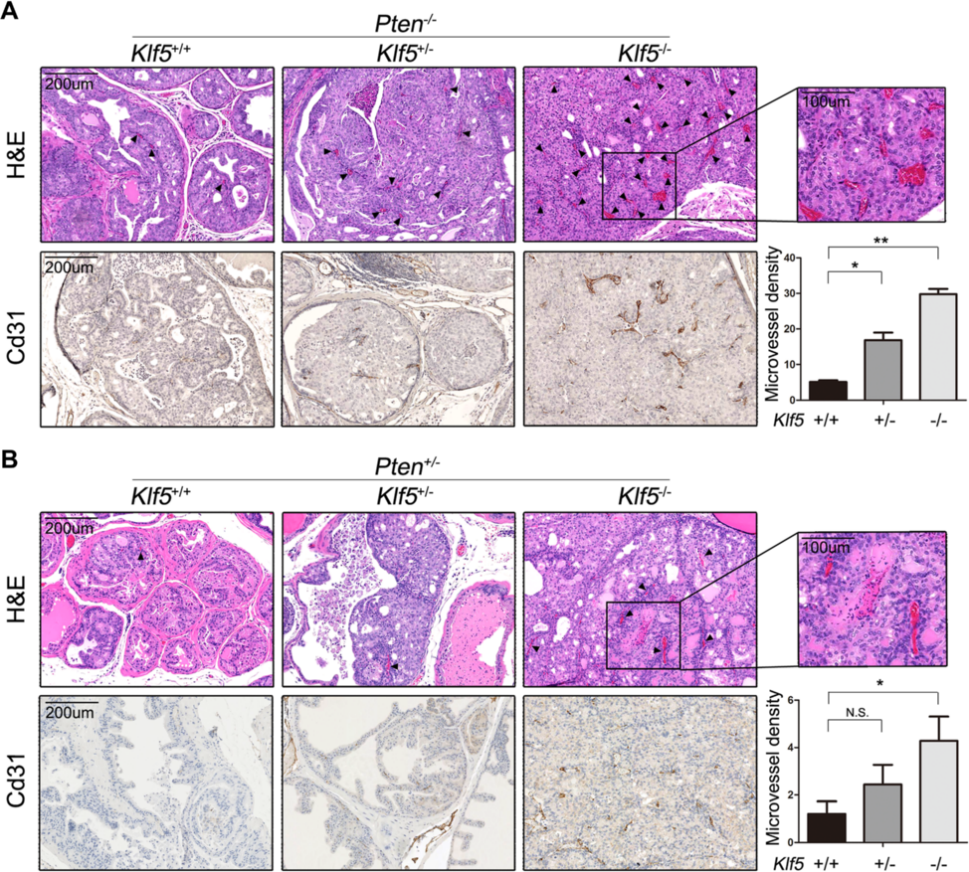
*Klf5* deletion increases blood microvessels in mouse prostate tumors. H&E staining and IHC staining of Cd31 for blood microvessels from prostates at 8 months for *Pten* ^−/−^ **(A)** and 12 to 18 months for *Pten* ^*+/−*^ **(B)**. Black arrows point to microvessels inside prostatic acini. Images at the right upper corner are higher magnifications of boxed areas. Microvessels were counted in dorsal lobes for *Pten* ^−/−^ prostates and the entire lobes for *Pten* ^+/−^ prostates. The total area of prostatic epithelia excluding the lumen space inside the acini was measured with ImageJ software. Gene status is indicated at the top of each panel, and + and – indicate wildtype and deletion of *Klf5* or *Pten*, respectively. Three to 6 mice were used for each group. Microvessel density was defined by the number of microvessels per mm^2^. *, P < 0.05; **, P < 0.01; N.S., not statistically significant.

To test whether *Klf5* deletion plays a role in tumor angiogenesis, we first examined H&E stained tissue sections for the number of intraepithelial blood vessels, indicated by histological appearance and the presence of red blood cells between wildtype tissues and those with *Klf5* deletion. We used dorsal prostate tumors of 8 month old mice for the *Pten*-null group, and whole prostate with high grade mPINs of 12 to 18 month old mice for the *Pten* hemizygous group. The number of microvessels was clearly increased by homozygous deletion of *Klf5* in both *Pten* groups (Figure 1A, 1B upper panels). To more accurately determine the number of microvessels in mPINs and prostate tumors, we performed immunohistochemical staining for the Cd31 endothelial cell marker and counted the number of microvessels indicated by positive Cd31 staining (Figure 1A, B, lower panels). The density of microvessels, determined by dividing the number of Cd31-marked microvessels by the total area of epithelial cells, was significantly increased by both hemizygous and homozygous deletions of *Klf5* in the *Pten*-null tumors and by *Klf5* homozygous deletion in the *Pten*-hemizygous group (Figure 1, bar figures). The increase in angiogenesis correlated with the increase in tumor mass and the decrease in *Klf5* expression caused by *Klf5* deletion in the same mice [9]. This result indicates that *Klf5* deletion promotes angiogenesis in both prostate tumors and mPINs induced by *Pten* deletion.

### *Klf5* deletion activates the angiogenesis transcriptional network and HIF1α in *Pten*-null mouse prostates

**Figure 2:**
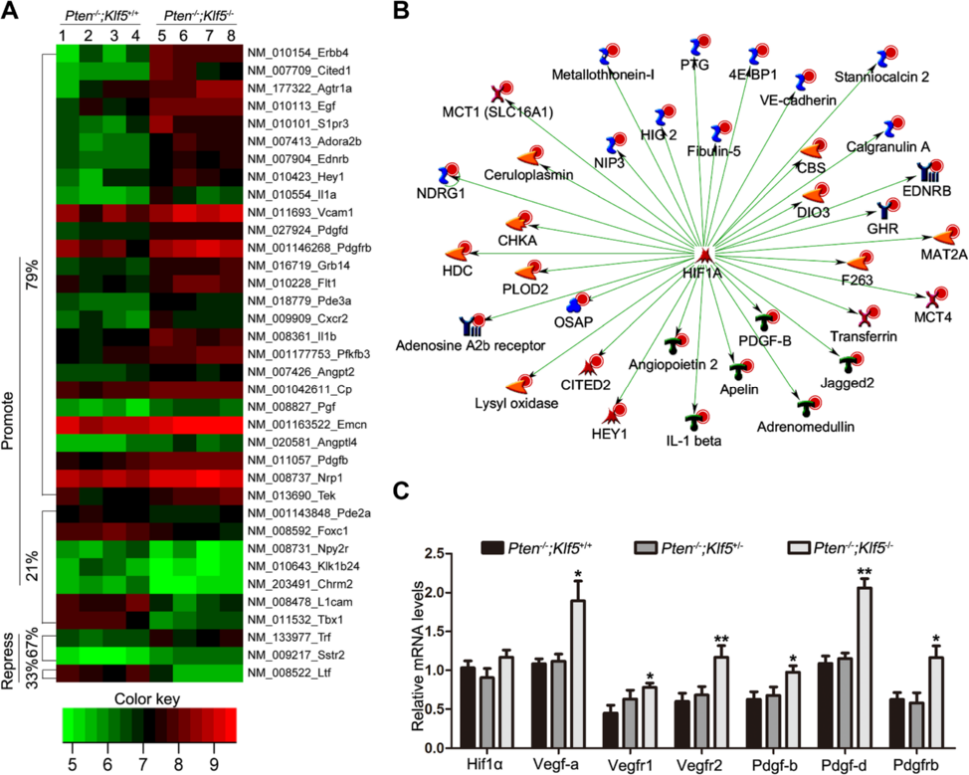
*Klf5* deletion dysregulates angiogenesis-related genes and enhances HIF1α transcriptional activity in *Pten*-null mouse prostates. **(A)** Heatmap of genes involved in angiogenesis based on the MetaCore analysis and PubMed publications and that had >1.5 fold change in expression between wildtype and *Klf5*-null mouse prostates (4 samples per group, indicated at the top). Genes are clustered by their effects on angiogenesis, with those promoting and repressing angiogenesis marked as “promote” and “repress”, respectively. Color intensities in the heat map represent log2 values of expression intensities. The percentage of upregulated or downregulated genes is shown. **(B)** HIF1α is activated by *Klf5* deletion, as indicated by the upregulation of its direct transcriptional target genes based on MetaCore’s interactome analysis for transcription factors. Different shapes of the nodes represent functional classifications of genes. Red dots on the upper right corner of each symbol indicate increased expression level, and green lines indicate positive regulation. **(C)** Detection of *Hif1α/Vegf* and *Pdgf* signaling molecules by real-time RT-PCR in 6-month-old *Pten*-null mouse dorsal prostates with indicated *Klf5* deletion status. Data for each genotype were from 8 mice.* and **indicate P < 0.05 and P < 0.01, respectively.

In our recent study, expression profiling and MetaCore analysis of mouse dorsal prostates at 6 months in the *Pten*-null background indicated that the process of blood morphogenesis is the most enriched upon the deletion of *Klf5* [9], composed of 39 differentially expressed genes. We searched the PubMed database for articles on each of the 39 genes, and reviewed these articles to determine whether the genes are pro- or anti-angiogenic. Among the 39 genes, 33 (84.6%) were reported to promote, 3 (7.7%) to suppress, and 3 (7.7%) to have unclear effects on angiogenesis. Twenty six (79%) of the 33 pro-angiogenic genes were significantly upregulated by *Klf5* deletion. The 36 genes with a promoting or repressing effect are listed in Additional file 1: Table S1, along with their expression fold changes caused by *Klf5* deletion, and a heatmap of these genes is shown in Figure 2A. Dysregulation of these genes indicates an enhanced angiogenesis transcription network resulting from *Klf5* deletion.

Based on the process indicative of active angiogenesis, we attempted to determine whether a specific pathway or a key transcription factor was activated by *Klf5* deletion to drive the pro-angiogenic phenotype, using the MetaCore program. Interestingly, HIF1α, an important mediator of tumor angiogenesis [16], was identified as a key transcription factor (Figure 2B) with *p*-value 4.2 × 10^−147^, as it affected the expression of 115 of the differentially expressed genes. Among the 115 genes, 49 were identified as direct transcriptional targets activated by HIF1α, and 34 of the 49 genes (69%) were upregulated by *Klf5* deletion (Figure 2B), suggesting an activated HIF1α transcription activity. VEGF and some of its related molecules are direct targets and functional mediators of HIF1α in angiogenesis [17,18], so we analyzed mRNA expression for several molecules of the HIF1α/VEGF signaling pathway, including *Hif1α*, *Vegf-a*, *Vegfr1* and *Vegfr2*, by real-time RT-PCR using 8 mouse dorsal prostates for each genotype of *Klf5* deletion in the *Pten*-null background. As shown in Figure 2C, the *Hif1α* mRNA level was unchanged, while *Vegf-a, Vegfr1* and *Vegfr2* mRNA levels were all increased by *Klf5* homozygous deletion. Of these, *Vegf-a* was not identified by the microarray analysis. *Klf5* deletion upregulated *Pdgf-b*, *Pdgf-d* and *Pdgfrb* mRNA expression in the microarray analysis (Figure 2A), suggesting an activated PDGF signaling pathway that could play an important role in angiogenesis [19]. *Pdgf-b* is also a transcriptional target of HIF1α [20] and could also be a functional mediator of HIF1α. Therefore, we examined mRNA expression of these PDGF signaling molecules by real-time RT-PCR, and confirmed the microarray analysis findings that *Pdgf-b*, *Pdgf-d* and *Pdgfrb* were upregulated by *Klf5* deletion (Figure 2C). Hemizygous deletion of *Klf5* had a mild effect on the expression of these molecules. These results indicate that *Klf5* deletion activates multiple key regulators of angiogenesis.

**Figure 3:**
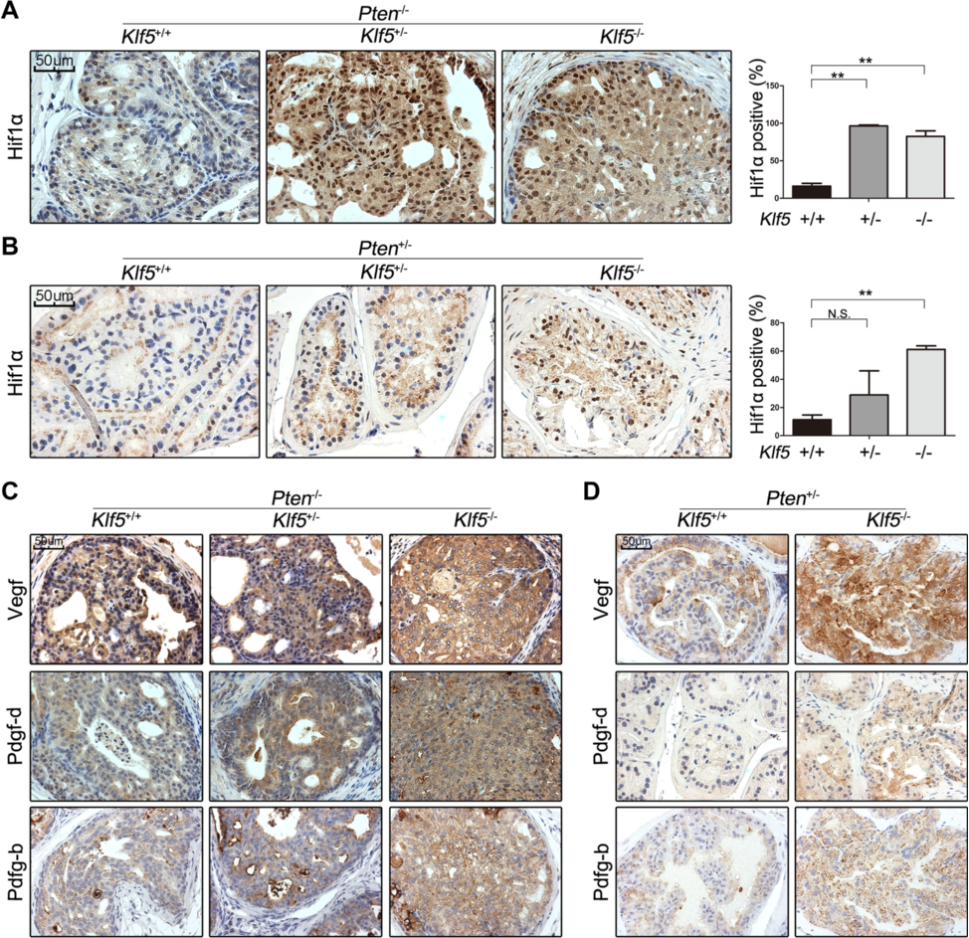
*Klf5* deletion increases protein expression of Hif1α and pro-angiogenic factors. **(A, B)** IHC staining of Hif1α in mouse prostates at 4 months for *Pten* ^−/−^ and 12 months for *Pten* ^+/−^. The numbers of Hif1α-positive cells and total prostatic epithelial cells were counted with ImageJ software for each mouse. **(C, D)** Protein expression of pro-angiogenic factors Vegf, Pdgf-d and Pdgf-b as detected by IHC staining with the same mouse tissues used in panels **A** and **B**. Three or 4 mice were used for each group. *, P < 0.05; **, P < 0.01; N.S., not statistically significant.

We also examined protein expression of Hif1α, Vegf, Pdgf-b and Pdgf-d in prostates with different *Klf5* deletion status by IHC staining. In *Pten*-null prostates, although the mRNA expression of *Hif1α* was not affected by *Klf5* deletion (Figure 2), its protein expression was increased in both the nucleus and the cytoplasm of prostatic cells by both hemizygous and homozygous deletions of *Klf5* (Figure 3A). In addition, almost all cells with *Klf5* deletion were positive for Hif1α expression in the nucleus (Figure 3A), which indicates an active Hif1α status [3]. In the context of *Pten* hemizygous deletion, accumulation of Hif1α was increased upon *Klf5* deletion in prostates of one year old mice, and homozygous deletion of *Klf5* also significantly increased the ratio of cells with positive Hif1α nuclear staining (Figure 3B). For pro-angiogenic factors Vegf, Pdgf-b and Pdgf-d, whose mRNA expression was upregulated by *Klf5* deletion (Figure 2A, C), an increase in their protein expression was also detected in *Pten*-null prostates with both hemizygous and homozygous deletions of *Klf5* (Figure 3C). In prostates with *Pten* hemizygous deletion, only homozygous *Klf5* deletion increased protein expression of these factors (Figure 3D). These results further indicate that *Klf5* loss induces the enhanced expression and activity of Hif1α accompanied with augmented expression of pro-angiogenic factors.

### Dysregulation of KLF5 in prostate cancer cells impacts tube formation and migration of HuVECs

**Figure 4:**
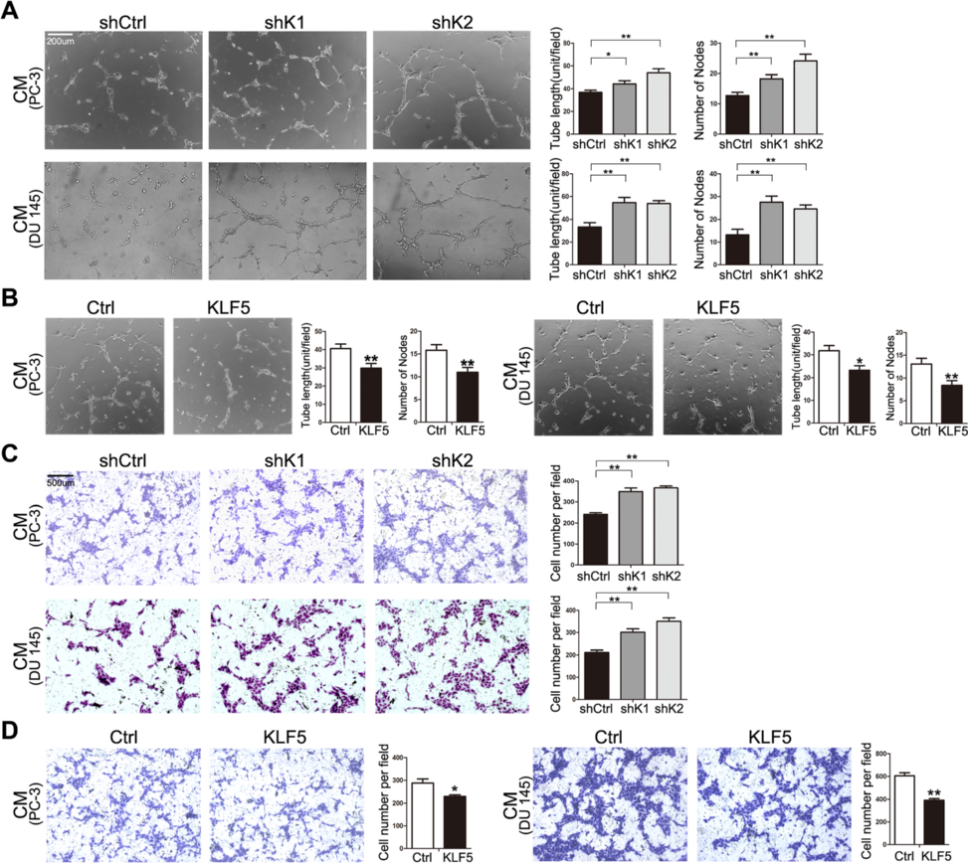
Expression changes in *KLF5* in human prostate cancer cell lines modulate the angiogenic activities of HuVECs. Tube formation assay **(A, B)** and transwell assay **(C, D)** of human umbilical vein endothelial cells (HuVECs) with conditioned media (CM) from PC-3 and DU 145 cells with *KLF*5 knockdown (A) and ectopic expression **(B)** mediated by lentiviral infection. For tube formation assay, tube length and node number from ten fields were measured with ImageJ software, and each unit is 100 pixels. For transwell assay, HuVECs were seeded in the upper chamber with serum-free medium. Conditioned media was added to the lower wells, and cells migrating to the bottom side of the upper chamber were stained with crystal violet and counted from 20 fields at 4× with ImageJ software. shK1 and shK2 indicate two shRNAs targeting *KLF5*(sh36 and sh37) **(A, C)**, and Ctrl and KLF5 indicate the pSin vector control and pSin-KLF5 expression construct, respectively **(B, D)**. *, P < 0.05; **, P < 0.01.

To further test the role of KLF5 in angiogenesis, we determined whether changes in *KLF5* expression in human prostate cancer cells directly modulate endothelial cell behaviors such as tube formation and migration, both of which are indicative of angiogenesis. We used PC-3 (*PTEN* null) and DU 145 (*PTEN* haploinsufficient) human prostate cancer cell lines [21] and human umbilical vein endothelial cells (HuVECs) for the analysis. *In vitro* cultured cells eliminate any effects of tumor growth *in vivo*, thus providing evidence for a direct effect of KLF5 on angiogenesis. We first knocked down *KLF5* using lentiviruses expressing shRNAs in PC-3 and DU 145 cells to mimic the KLF5 and PTEN status in the mouse model. Using stable cell populations with efficient knockdown of *KLF5*, we collected conditioned media and applied them to HuVECs for tube formation assay, in which HuVECs formed a tubular network of interconnecting branches after 5–7 hours. Knockdown of *KLF5* in both PC-3 and DU 145 cell lines significantly increased the cumulative tube length and the number of tube nodes in HuVECs (Figure 4A). Considering that both PC-3 and DU 145 cell lines express a relatively lower level of KLF5 [22,23], we also overexpressed *KLF5* by lentiviral infection in these two cell lines and performed HuVEC tube formation assay with conditioned media. Consistent with the knockdown result, ectopic expression of *KLF*5 significantly inhibited tube formation *in vitro*, as indicated by the cumulative tube length and the number of tube nodes (Figure 4B).

Vascular endothelial cell migration is a critical step and another indicator of angiogenic ability [24], so we also performed transwell and wound healing assays to evaluate the effect of *KLF5* dysregulation on the migration of HuVECs. Conditioned media from both PC-3 and DU 145 cell lines with *KLF5* knockdown significantly promoted, whereas those from these cells with ectopic expression of *KLF5* significantly inhibited, the migration of HuVECs, as indicated by the number of cells migrated through the membrane in the transwell assay (Figure 4C, D) and the extent of gap in the wound healing assay (Additional file 2: Figure S1). These *in vitro* assays provide another line of evidence for the role of KLF5 in angiogenesis in *PTEN*-deficient prostate cancer cells.

### KLF5 also regulates the expression of HIF1α and other pro-angiogenic factors in human prostate cancer cells

**Figure 5:**
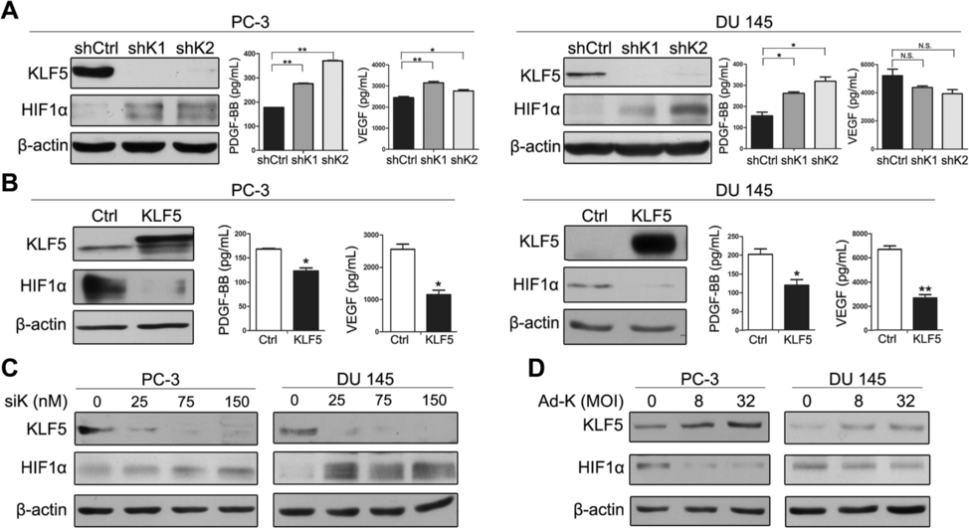
KLF5 decreases protein expression of HIF1α and secretion of pro-angiogenic factors. In PC-3 and DU 145 cells with stable knockdown **(A)**, ectopic expression **(B)**, RNAi-mediated transient knockdown **(C)**, or adenovirus-mediated overexpression **(D)** of *KLF5*, HIF1α protein was detected by Western blotting whereas secretory pro-angiogenic factors PDGF-BB and VEGF in the conditioned media were detected by ELISA. shK1 and shK2 indicate 2 lentiviral shRNAs targeting *KLF5* (sh36 and sh37), siK represents siRNA targeting *KLF5* (siKLF5), and Ad-K represents adenovirus expressing KLF5. *, P < 0.05; **, P < 0.01; N.S., not statistically significant.

Based on our finding that manipulation of *KLF5* expression in human prostate cancer cells affected tube formation and migration of HuVECs (Figure 4), we further tested whether manipulation of *KLF5* expression also affects the expression of HIF1α and other pro-angiogenic factors as in mouse tissues. We performed Western blotting to detect HIF1α expression and ELISA to detect the secretion of VEGF and PDGF-B in prostate cancer cells, whereas PDGF-D is undetectable by ELISA due to a low level of expression. As shown in Figure 5A, knockdown of *KLF*5 in PC-3 and DU 145 cells by lentiviral shRNA clearly increased the expression of HIF1α and PDGF-B, although the expression of VEGF was increased in PC-3 cells but slightly decreased in DU 145 cells (not statistically significant). Consistently, ectopic expression of *KLF5* by lentiviral infection in the same two cell lines decreased the expression of HIF1α, VEGF and PDGF-B (Figure 5B). We also used transient transfection of siRNAs to knock down *KLF5* (Figure 5C) and adenoviral infection to transiently express *KLF5* (Figure 5D) in the two prostate cancer cell lines, and confirmed that knockdown and overexpression of *KLF5* respectively increased and decreased the expression of HIF1α (Figure 5C, D).

We also detected mRNA expression of *HIF1α*, *VEGF* and *PDGF-B* in human prostate cancer cells with altered *KLF5* expression. *PDGF-B* and *VEGF-A* mRNA showed the same trend of change as their protein expression (Additional file 3: Figure S2), consistent with the results from mouse tissues (Figures 2 and 3). *HIF1α* mRNA expression also responded to *KLF5* manipulation and showed the same trend of expression change as its protein expression in PC-3 cells (Additional file 3: Figure S2 left panel), in contrast to the result from mouse tissues where no expression change of *HIF1α* mRNA was detectable (Figure 2C).

### KLF5 loss induces HIF1α protein accumulation by activating AKT signaling

Following the detection of the angiogenic phenotype and the activation of the HIF1α-driven pro-angiogenic molecular network upon KLF5 loss in both mouse tissues and cultured human prostate cancer cells, the fundamental question became how KLF5 loss activates the HIF1α network to promote angiogenesis. Considering that the expression change of HIF1α upon KLF5 loss mainly occurred at the protein level but not the mRNA level (Figures 2, 3 and 5) and that post-transcriptional regulation is the major mechanism of HIF1α regulation [3], we focused on the hypothesis that KLF5 loss activates HIF1α post-transcriptionally.

**Figure 6:**
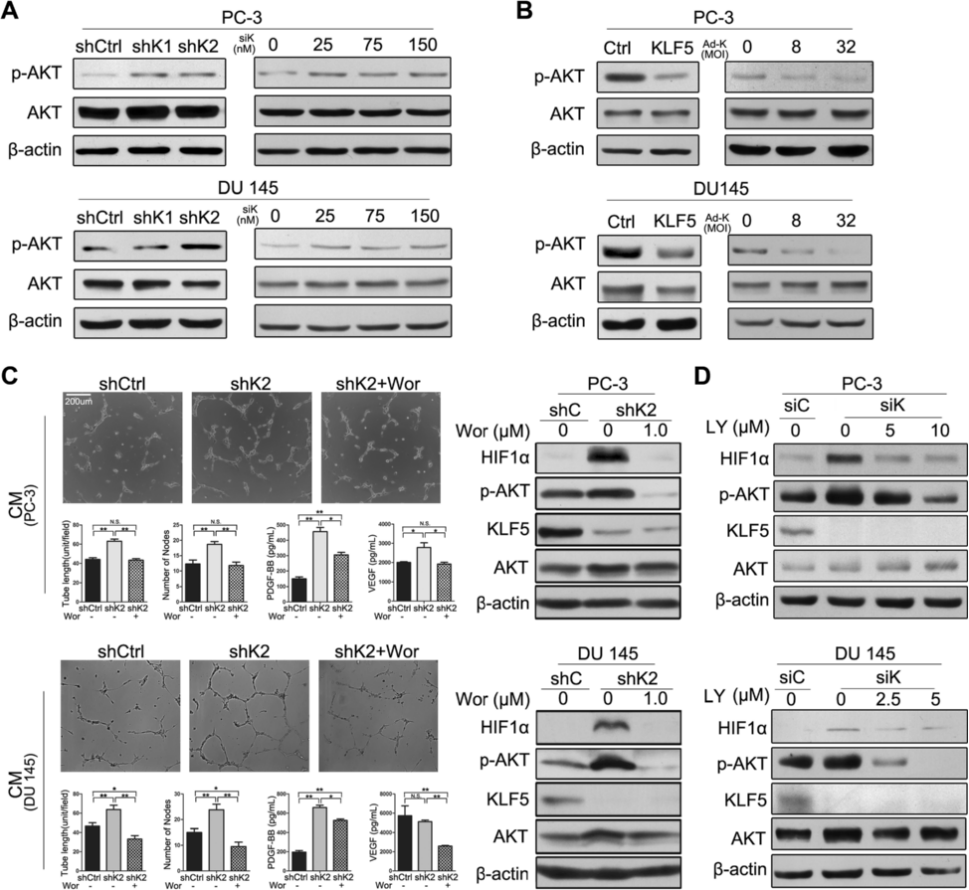
KLF5 regulates HIF1α protein via AKT1 kinase. **(A, B)** Phospho-AKT (S473) is upregulated by the knockdown of *KLF5* **(A)** and downregulated by ectopic expression of *KLF5* **(B)** in PC-3 and DU 145 human prostate cancer cells, as detected by Western blotting. **(C)** Wortmannin, a PI3K inhibitor, rescued the elevation of HuVEC tube formation and the expression of p-AKT, HIF1α, PDGF-B and VEGF induced by *KLF*5 knockdown. Conditioned media, collected after 24 hours of 1 μM Wortmannin treatment or solvent control from PC-3 and DU 145 cells with *KLF*5 knockdown (shK2), were used for HuVEC tube formation assay and detection of PDGF-BB and VEGF expression by ELISA. Expression of HIF1α and p-AKT was detected by Western blotting. **(D)** Detection of HIF1α and phospho-AKT (S473) by Western blotting in PC-3 and DU 145 cells with transient knockdown of *KLF*5 and treatment of PI3K inhibitor LY294002 for 3 hours. Solvent control for Wortmannin and LY294002 was DMSO. shK1 and shK2 indicate 2 lentiviral shRNAs targeting *KLF5* (sh36 and sh37), siK represents siRNA targeting *KLF5* (siKLF5), and Ad-K represents adenovirus expressing KLF5. The concentration of siRNA was at 100 nM for panel D. *, P < 0.05; **, P < 0.01; N.S., not statistically significant.

In our recent study, we found that *Klf5* deletion activates multiple oncogenic signaling pathways including the PI3K/AKT, ERK and EGF/EGFR pathways [9]. On the other hand, it has been well established that AKT and ERK activation leads to the accumulation of HIF1α mainly by either translational activation or stabilization of HIF1α [25-29]. We therefore tested whether KLF5 loss induces HIF1α protein accumulation by activating AKT and ERK signaling. Consistent with the finding from mouse prostates [9], Western blotting demonstrated that both stable and transient knockdown of *KLF5* in PC-3 and DU 145 cells increased, and ectopic expression of *KLF5* decreased, the expression of activated AKT (p-AKT) (Figure 6A, B). For both EGFR and ERK, activity changes were inconsistent between human cancer cell lines and mouse tissues. Specifically, EGFR activation was slightly downregulated by both *KLF5* knockdown and ectopic expression in the two cell lines, while ERK activation was downregulated upon *KLF5* ectopic expression only in PC-3 cells but not in DU 145 cells. ERK activity was unchanged when *KLF5* was knocked down in both the PC-3 and DU 145 cell lines. This result suggests that EGFR and ERK are less crucial than AKT in mediating the effect of KLF5 on HIF1α (Additional file 4: Figure S3). Expression changes of p-AKT (Figure 6A, B) corresponded nicely to those of HIF1α in the same cells (Figure 5).

To further test whether AKT activation mediates HIF1α accumulation induced by KLF5 loss, we treated cells with Wortmannin, an irreversible PI3K/AKT inhibitor. We found in both PC-3 and DU 145 cells that inhibition of AKT activity by Wortmannin prevented the enhancement of tube formation and the upregulation of HIF1α, PDGF-B and VEGF by *KLF5* knockdown (Figure 6C). Similarly, treatment of PC-3 and DU 145 cells with the LY294002 PI3K inhibitor upon *KLF*5 transient knockdown also abolished the upregulation of HIF1α protein (Figure 6D).

## Discussion

### *Klf5* loss enhances angiogenesis in prostatic tumorigenesis

In this study, we performed histological and molecular analyses in mouse prostate tumors and human prostate cancer cells with the deletion or knockdown of *KLF5* and *PTEN*, and obtained multiple lines of evidence suggesting the role of KLF5 loss in tumor angiogenesis. First of all, *Klf5* loss enhanced angiogenesis not only during prostatic tumorigenesis initiated by homozygous deletion of *Pten* (Figure 1A) but also in mPINs induced by hemizygous deletion of *Pten* (Figure 1B), and *Klf5*’s homozygous deletion was more potent than its hemizygous deletion in the increase of microvessels (Figure 1). Secondly, conditioned culture media from human prostate cancer cells with deficient or insufficient *PTEN* and *KLF5* knockdown increased tube forming capacity and migration of HuVECs (Figure 4), which are indicative of angiogenic potential. Overexpression of *KLF5* in human prostate cancer cells showed a consistent effect on HuVECs (Figure 4). Thirdly, at the molecular level *Klf5* deletion significantly elevated the expression of HIF1α and its pro-angiogenic functional effectors such as VEGF and PDGF (Figures 2, 3 and 5), which represent the most potent inducers of tumor angiogenesis. Our findings are consistent with a previous study in which *Klf5* deletion in mouse corneas enhanced neovascularization [12], indicating that KLF5 loss in some epithelial tissues such as prostate and cornea promotes angiogenesis.

While KLF5 loss enhances angiogenesis, the extent of enhancement may depend on tumorigenesis including the development of cancer precursors such as mPIN, because deletion of *Klf5* alone, which does not induce neoplastic alterations in mouse prostates [30], did not affect angiogenesis and HIF1α expression (data not shown). On the other hand, other genetic alterations combined with *Pten* deletion more often do not affect angiogenesis in mouse prostate tumors. For example, overexpression of COUP-TFII in *Pten*–null prostates results in aggressive metastasis-prone prostate tumors but does not alter angiogenesis [31]. In addition, there is no evidence for alterations in angiogenesis when other tumor suppressor genes such as *p53*, *Smad4*, *Nkx3-1* and *p27* are deleted in *Pten*-null mouse prostates [32-35].

Our recent study showed that interruption of KLF5 acetylation also promotes prostate cancer growth [8]. However, tumor promotion by unacetylated KLF5 does not appear to affect angiogenesis, as the number of microvessels and the expression of HIF1α and VEGF were not affected by changes in the acetylation status of KLF5 (data not shown), and angiogenesis was not among the affected processes based on bioinformatic analysis [8].

### KLF5 loss enhances angiogenesis partially by upregulating HIF1α and its downstream pro-angiogenic factors including VEGF and PDGF

In understanding how KLF5 loss enhances tumor angiogenesis, our Gene Ontology (GO) enrichment analysis of differentially expressed genes upon *Klf5* deletion identified blood morphogenesis as the most affected biological process, involving a molecular signature of activated pro-angiogenic genes (Figure 2A). In addition, network analysis of the differentially expressed genes led to the identification of HIF1α, a crucial inducer of angiogenesis, as a key transcription factor activated by *Klf5* deletion, which transcriptionally activated 34 genes upon *Klf5* deletion (Figure 2B). Among the 34 genes, secretory protein Pdgf-b is a well-established transcriptional target of HIF1α and a crucial regulator of angiogenesis [2,20] (Figure 2C). Although VEGF, another key angiogenesis regulator and secretory protein induced by HIF1α [17], was not identified by the microarray analysis as being upregulated by *Klf5* deletion, our expression analyses demonstrated that KLF5 loss indeed upregulated HIF1α at the protein level and VEGF and PDGF at both the mRNA and protein levels in both mouse prostate tumors and human prostate cancer cells with deficient or insufficient PTEN (Figure 2, 3 and 5).

In addition to the well-established angiogenesis regulators HIF1α, VEGF and PDGF [36], other molecules could also be involved in enhanced angiogenesis after KLF5 loss. For example, Apelin and Adora2b, both of which are transcriptional targets of HIF1α, were upregulated by *Klf5* deletion (Figure 2B) and have been shown to promote angiogenesis in certain types of cells and animal models [37,38]. Another group among the 36 genes that play a role in angiogenesis and were upregulated by *Klf5* deletion in mouse prostates (Figure 2A, Additional file 1: Table S1) included *Il1* and *Ang*, both encoding secretory proteins in prostatic epithelial cells that could also mediate the role of KLF5 loss in angiogenesis.

### Upregulation of HIF1α by KLF5 loss is likely mediated by the activation of PI3K/AKT signaling

HIF1 consists of two subunits, the α unit, which is induced by hypoxia, and the β unit, which is expressed constitutively [16]. Regulation of HIF1α mainly occurs at the protein level, since it is degraded by the ubiquitin proteasome pathway (UPP) under normoxia conditions but is stabilized under hypoxia conditions in a tumor environment [2,3]. HIF1α can also be upregulated independent of hypoxia upon the activation of growth factor-dependent signaling pathways [3]. For example, forced PTEN overexpression reduces HIF1α expression, whereas activated PI3K/AKT signaling upregulates HIF1α expression [28,29]. Our recent study established that *Klf5* deletion leads to the activation of PI3K/AKT and ERK signaling in mouse prostate tumors [9]. Using cultured human prostate cancer cell lines with deficient or insufficient PTEN, not only did we confirm the activation of AKT by KLF5 loss (Figure 6A), we also found that activated AKT indeed mediated the upregulation of HIF1α by KLF5 loss, since treatment with PI3K inhibitors prevented both the activation of AKT and the accumulation of HIF1α (Figure 6C, D). Treatment with PI3K inhibitors also prevented the enhancement of tube formation by KLF5 loss (Figure 6C). Therefore, one mechanism for the upregulation of HIF1α by KLF5 loss in PTEN deficient or insufficient prostate cancer cells appears to be the activation of AKT. Previous studies have demonstrated that PI3K/AKT signaling promotes HIF1α protein accumulation by either increasing its translation or by inhibiting its degradation or both [25,39,40]. Currently it is unknown which process is most involved in HIF1α accumulation induced by KLF5 loss and subsequent PI3K/AKT activation.

It is well known that PTEN is a lipid phosphatase whose loss activates PI3K/AKT signaling [41], which is related to HIF1α upregulation and enhanced angiogenesis in some human cancer cells [42,43]. Our recent study demonstrated that Klf5 loss in *Pten*-null prostate tumors also activates PI3K/AKT signaling by upregulating multiple extracellular growth factors (e.g. EGF), cytokines and their receptors [9]. Deletion of *Klf5* alone in mouse prostates did not affect angiogenesis (data not shown), and neither did *Pten* deletion alone in mouse prostates cause apparent angiogenesis [13]. Therefore, KLF5 loss and PTEN loss had an additive effect on the activation of PI3K/AKT signaling [9], HIF1α accumulation (Figure 6), and angiogenesis (Figure 1). The precise function of KLF5 in mediating AKT activation is still under investigation.

While two other oncogenic signaling pathways, EGF/EGFR and ERK, were activated in mouse prostate tumors upon *Klf5* deletion [9], and KLF5 could positively regulate EGFR expression and ERK activation [44,45], their changes in activity in response to KLF5 modulation were inconsistent between human prostate cancer cell lines PC-3 and DU 145 and mouse prostate tissues (Additional file 4: Figure S3). Therefore, whereas EGFR and ERK activation may still play a role in the upregulation of HIF1α by *Klf5* deletion in mouse prostate tumors, they are less crucial for KLF5 to regulate HIF1α in human cancer cell lines PC-3 and DU 145.

There are other mechanisms that could account for how KLF5 regulates HIF1α protein, including transcriptional regulation, miRNA and hypoxia. In colon and lung cancer cells, KLF5 was shown to directly regulate the transcription of *HIF1α* [46,47]. However, our results (Figure 2 & Additional file 3: Figure S2) showed that KLF5 did not alter the transcription of *HIF1α* either in mouse prostates or in human prostate cancer cells. HIF1α can also be regulated by miRNAs including miR-138 [48]. While miR-138 appeared to be downregulated by *Klf5* deletion in mouse prostate tumors, it was unaltered by KLF5 loss in human prostate cancer cells (data not shown). Hypoxia is a potent inducer of HIF1α, but it did not appear to be necessary for KLF5 loss to induce HIF1α, at least in cancer cell lines, because the morphological and molecular alterations in PC-3 and DU 145 cells were detected under normoxic conditions (Figures 4, 5 and 6). Nevertheless, it remains to be clarified whether these additional mechanisms are involved in the HIF1α accumulation induced by KLF5 loss.

### Enhanced angiogenesis by KLF5 loss may partially account for its tumor promoting function and have implications in human prostate cancer

Our recent study demonstrated that *Klf5* deletion promotes mouse prostate tumorigenesis initiated by *Pten* deletion, as indicated by the accelerated emergence and progression of mPIN caused by Pten insufficiency and the more severe morphological and molecular abnormalities of prostate tumors caused by Pten deficiency [9]. In that study, enhanced cell proliferation was identified as a mechanism for the tumor promoting effect of *Klf5* deletion [9]. Our current study suggests that enhanced angiogenesis by KLF5 loss is likely another mechanism for the tumor promoting effect of KLF5 loss during prostatic tumorigenesis initiated by other oncogenic factors, which is in addition to its previously reported promoting function in tumor cell proliferation [9].

During the development of human prostate cancer, chromosome 13q is one of the most frequently deleted chromosomes, and the deletion involves multiple regions and genetic foci in 13q14 and 13q21, including *RB1* and *FOXO1* at 13q14 and *KLF5* at 13q21 [22,49-52]. Whereas *RB1* is a well-established tumor suppressor gene at 13q14, the tumor suppressor function of *KLF5* has also been demonstrated in both xenograft and knockout models [7,9]. In addition, conventional comparative genomic hybridization (CGH) studies indicate that deletion spanning the *KLF5* locus could occur in as much as 39% of prostate cancers [6], and our earlier deletion analyses suggest that hemizygous deletion is more common than homozygous deletion in prostate cancer [22,50]. In the latest genome-wide copy number change studies, *KLF5*’s deep deletion, which likely indicates homozygous deletion, has been detected in 10% of prostate cancers in The Cancer Genome Atlas (TCGA) database (tcga-data.nci.nih.gov) and in 20% of metastatic prostate cancers in another database [53], as analyzed by the cBioPortal program [54,55]. Hemizygous deletion induces haploinsufficiency of *KLF5* [9,10], and our recent publication demonstrated that hemizygous deletion of *Klf5* in mouse prostates indeed promotes tumor development [30]. Therefore, frequent hemizygous and homozygous deletions induce frequent insufficiency and deficiency, respectively, of KLF5 during prostatic carcinogenesis. Genetic amplification and overexpression of the WWP1 E3 ubiquitin ligase, which degrades KLF5 protein, also frequently occur in human prostate cancer [56,57], causing excess protein degradation and insufficiency of KLF5. Therefore, enhanced tumor angiogenesis represents another mechanism for how KLF5 insufficiency and deficiency could promote the development and progression of human prostate cancer.

As in many other types of cancers, prostatic carcinogenesis depends on the accumulation of multiple genetic and epigenetic alterations, and deletions of *KLF5* and *PTEN* can occur in the same tumors [22,58-61]. For example, 7 of the 25 (28%) prostate cancers with *KLF5* deep deletion in the TCGA database and 4 of the 11 (36%) metastatic prostate cancers in another database also have *PTEN* deep deletion or truncating mutation [53], as revealed by the cBioPortal analysis [54,55]. Further related to our current findings, it has been reported that *PTEN* inactivation is positively associated with higher microvessel densities in clinically localized prostate cancer [43], and PTEN expression is negatively associated with VEGF expression in gastric carcinomas [62]. Another study demonstrated that in high-grade prostate adenocarcinomas, 46% of them showed higher nuclear HIF1α immunostaining, and 89% expressed higher levels of VEGF [63]. In the context of concurrent deletions of *KLF5* and *PTEN* in human prostate cancer, our findings further suggest that during the development of human prostate cancer, KLF5 loss and PTEN loss cooperate to enhance tumor angiogenesis by upregulating HIF1α and VEGF, which remains to be examined.

In summary, we found that deletion of *Klf5* promoted angiogenesis in *Pten* deletion-initiated mouse prostate tumors. Promotion of angiogenesis by Klf5 loss involved the upregulation of pro-angiogenic molecules including HIF1α and its downstream targets VEGF and PDGF. Upregulation of HIF1α was mediated at least in part by the activation of PI3K/AKT oncogenic signaling. Consistently, knockdown of *KLF5* in PTEN-insufficient human prostate cancer cells increased tube formation and migration in HuVECs, enhanced PI3K/AKT activity, and upregulated HIF1α, VEGF and PDGF. These findings indicate that *KLF*5 loss enhances angiogenesis during prostatic carcinogenesis initiated by other oncogenic factors, and that enhanced angiogenesis could be a mechanism for the tumor promoting function of KLF5 loss.

## Materials and methods

### Cell lines, siRNAs, inhibitors and antibodies

Human umbilical vein endothelial cells (HuVECs) were purchased from Life Technologies and cultured in Medium 200 with low serum growth supplement (LSGS) (Carlsbad, CA). PC-3 and DU 145 human prostate cancer cell lines and the 293 T human embryonic kidney cell line were purchased from American Type Culture Collection (ATCC) (Manassas, VA), and propagated following ATCC’s instructions.

SiRNA against *KLF5* (SiKLF5), developed in a previous study [64] with the sequence of AAGCUCACCUGAGGACUCATT, was used to knock down *KLF5* in human cells at a concentration of 150 nM or as indicated in related figures. The control siRNA (siCtrl), AAUUCUCCGAACGUGUCACGUTT, was purchased from Thermo Fisher (Waltham, MA) used to bring the siRNA concentration to the same in concentration gradient RNAi assay.

PI3K inhibitors Wortmannin and LY294002 were purchased from Cell Signaling Technology (Danvers, MA), and dissolved in dimethyl sulfoxide (DMSO).

Antibodies used in this study included the following: anti-HIF1α and anti-PDGF-B from Novus Biologicals (Littleton, CO), anti-PDGF-D from Santa Cruz (Santa Cruz, CA), anti-phospho-AKT (S473), anti-AKT, anti-phospho-EGFR (Y1068), anti-EGFR, anti-phospho-ERK1/2 and anti-ERK1/2 from Cell Signaling Technology, anti-VEGF and anti-CD31 from Abcam (Cambridge, MA), and anti-β-actin from Sigma. The antibody against KLF5 has been described previously [23].

### Mouse prostate tissues with different *Klf5* and *Pten* deletion status

All mouse prostate tissues with different *Klf5* and/or *Pten* deletion status, including prostate tumors, mouse prostatic intraepithelial neoplasia (mPIN) and control tissues of different ages, were from our recently published study [9].

### Production of lentiviruses and adenoviruses and their infection of cells

Lentiviruses expressing *KLF5* and shRNA targeting *KLF5* were produced by transfecting respective plasmids in 293 T cells following the protocols described on the Addgene website (http://www.addgene.org/lentiviral/protocols-resources/). The PLKO.1 expression vector was used for shRNA expression, and the 2 shRNAs targeting *KLF5*, sh36 and sh37, were described in our previous study [65]. The pSin expression vector was used for *KLF5* expression (pSin-KLF5) as in our previous study [66]. Following our previously described procedures [65], viruses were added to culture media to infect PC-3 and DU 145 cells, and medium containing 1 μg/ml puromycin was used to select infected cells for at least 96 hours to obtain populations for analysis.

Adenoviruses expressing *KLF5* (Ad-KLF5) and GFP (Ad-GFP) were prepared and used following our established procedures [64]. MOI (multiplicity of infection) was defined as the average number of virus particles infecting a cell.

### RNA isolation, real-time RT-PCR and microarray expression analysis

Mouse dorsal prostates or tumor tissues were dissected freshly and stored in liquid nitrogen, and total RNA was isolated by using the RNeasy Mini Kit (Qiagen, Valencia, CA) following the manufacturer’s instructions. TRIzol reagent (Life technologies) was used for isolating total RNA from cultured cellsfollowing previously established procedures [67]. First-strand cDNA was synthesized from total RNA using a Promega reverse transcription kit (Madison, WI).

Real-time RT-PCR was performed using the SYBR Premix reagent (Takara, Tokyo, Japan) and ABI Fast 7500 Real-time PCR System (Applied Biosystems, South San Francisco, CA). The 2^(−△△Ct)^ method was used to calculate relative fold changes with *GAPDH* as the internal control. Primers used for real-time RT-PCR are listed in Additional file 5: Table S2.

Genes with significantly different expression between *Klf5* wildtype and *Klf5*-null 6-month old prostate tumors in the context of *Pten* deletion, as described in our recent study [9], were uploaded into the web-based MetaCore platform (http://thomsonreuters.com/metacore/), and angiogenesis-related genes were enriched using Gene Ontology (GO)-based functional process enrichment. Whether a gene functions in angiogenesis was further evaluated by literature review among PubMed publications with “angiogenesis” and a gene’s name as key words. The algorithm of “activated transcription factor regulating genes in the experimental data” was used to build a HIF1α-centered network with those that were not only direct transcriptionally activated targets of HIF1α but were also upregulated by *Klf5* deletion.

### Immunohistochemistry (IHC), Western blotting and enzyme-linked immunosorbent assay (ELISA)

IHC staining was performed following our established protocols [30] and nuclei were stained with hematoxylin. Mounted slides were scanned with the Hamamatsu NanoZoomer scanner (Hamamatsu Corporation, Bridgewater, NJ) or photographed with a microscope. When needed, cell numbers were counted by using the ImageJ program to calculate the ratio of positive cells for a protein. Western blotting was performed following our previously established procedures [68]. For secretory factors, conditioned culture media were collected from 100% confluent cells in 100-mm dishes, and commercial ELISA kits against VEGF and PDGF-BB from Abcam were used to determine the expression level of VEGF and PDGF-BB following the manufacturer’s instructions.

### Tube formation assay in HUVECs

Prostatic epithelial cells were seeded in 100-mm dishes, grown to 90% confluence at 48 hours, washed with PBS twice, and then cultured in 3 ml of medium containing 1% serum for another 24 hours to reach 100% confluence. Conditioned media were then collected, centrifuged at 1500 rpm/min, and the supernatant was used for experiments as previously described [69]. A total of 3 × 10^4^ HuVECs were seeded into each well of a 48-well plate coated with growth-factor reduced Matrigel (BD Biosciences, San Jose, CA). After culturing for 5 and 8 hours with conditioned media or the HuVEC culture medium, the capillary-like structures were captured with the Olympus IX51 microscope, and the extent of tube formation was assessed by measuring the cumulative tube length and counting the number of tube nodes (the intersection among 3 or more tubes) in a 10× field using ImageJ software. At least 10 fields were examined to determine cell tube formation ability [70].

### Wound healing assay in HuVECs

Confluent monolayer HuVECs cultured in 24-well plates were scratched with the tip of a 10-μl pipette tip to generate a cross wound, washed with PBS twice, and cultured with conditioned media for another 13–15 hours to allow the gap to close. Photographs of gaps at 0 and 13–15 hours were taken with an Olympus IX51 inverted microscope at 10× magnification, and the extent of wound closure was assessed by calculating the ratio of the initial to the final wound area with Image J software. At least 4 fields were examined to determine cell migration ability [71].

### Transwell migration assay in HuVECs

Five x 10^4^ HuVECs in 200 μl serum-free medium were seeded in the upper chamber of an insert (BD Biosciences) in a 24-well plate, and 800 μl of conditioned medium was added to the lower chamber. After 15–17 hours, cells in the insert were fixed in 4% paraformaldehyde (Sigma) and stained with 0.1% crystal violet (Sigma). Cells inside the transwell chamber were scraped with a cotton swap, while cells outside the chamber were photographed with an inverted microscope. The number of cells per field at 4× magnification was counted with Image J software, and at least 10 fields were examined to determine cell migration ability [65].

### Statistical analysis

Results from real-time RT-PCR and ELISA were expressed as means ± standard derivation, while readings from other experiments were expressed as means ± standard error. The statistical significance for a difference between two groups was determined by unpaired Student *t* test. A *P*-value of 0.05 or smaller was considered statistically significant.

## Additional files

Additional file 1: Table S1. Differentially expressed genes involved in angiogenesis, as identified by the MetaCore program. Fold change between *Klf5*-wildtype and *Klf5*-null groups are shown for each of the genes. P and R indicate promoting and repressive functions, respectively, of a gene in angiogenesis. Additional file 2: Figure S1. Expression changes in *KLF5* in PC-3 and DU 145 prostate cancer cells modulate wound healing in HuVECs. Confluent HuVECs cultured in conditioned media were scratched with a pipette tip and photographed from 4 fields at 0 and 13-15 hours for each sample. shK1 and shK2 indicate two shRNAs targeting *KLF5* (sh36 and sh37) (A), Ctrl and KLF5 indicate lentiviruses expressing the pSin vector control and KLF5, respectively (B). Areas of wound were measured by using ImageJ software to indicate the extent of wound closure. *, P<0.05; **, P<0.01. Additional file 3: Figure S2. Modulation of *KLF5* expression affects mRNA expression of pro-angiogenic factors. Real-time RT-PCR was used to detect mRNA expression of *HIF1α*, *VEGF-A*, *PDGF-B* and *PDGF-D* mRNA in PC-3 and DU 145 prostate cancer cells with stable knockdown (A) or ectopic expression (B) of *KLF5*. shK1 and shK2 indicate two shRNAs targeting *KLF5* (sh36 and sh37) (A)*,* Ctrl and KLF5 indicate pSin vector control and pSin-KLF5 (B), respectively. *, P<0.05; **, P<0.01. (TIFF 334 kb) Additional file 4: Figure S3. EGFR and ERK activation do not appear to mediate the upregulation of HIF1α by *KLF5* knockdown in human prostate cancer cell lines. Protein expression of p-EGFR (Y1068), EGFR, p-ERK and ERK were determined by Western blotting in PC-3 and DU 145 cells with stable knockdown or ectopic expression of *KLF5*. shK1 and shK2 indicate two shRNAs targeting *KLF5* (sh36 and sh37), and Ctrl and KLF5 indicate the pSin vector control and pSin-KLF5 expression construct, respectively. Additional file 5: Table S2. Primer sequences used in this study.

## Abbreviations

KLF5: Krüppel-like factor 5
HIF1α: Hypoxia-inducible factor 1-alpha
PTEN: Phosphatase and tensin homolog
PI3K: Phosphatidylinositol-4,5-bisphosphate 3-kinase
ERK: Extracellular signal-regulated kinases
MAPK: Mitogen-activated protein kinase
EGF: Epidermal growth factor
VEGF: Vascular endothelial growth factor
PDGF: Platelet-derived growth factor
HuVEC: Human umbilical vein endothelial cells
IHC: Immunohistochemistry
ELISA: Enzyme-linked immunosorbent assay
GO: Gene ontology
