# Supplementary material for: KLF5 inhibits angiogenesis in PTEN-deficient prostate cancer by attenuating AKT activation and subsequent HIF1α accumulation
Source: Mol Cancer. 2015 Apr 21;14:91. doi: 10.1186/s12943-015-0365-6 (PMC4417294; doi:10.1186/s12943-015-0365-6)
Supplement: Additional file 1: Table S1. — Differentially expressed genes involved in angiogenesis, as identified by the MetaCore program. Fold change between Klf5-wildtype and Klf5-null groups are shown for each of the genes. P and R indicate promoting and repressive functions, respectively, of a gene in angiogenesis. [file 12943_2015_365_MOESM1_ESM.docx]

| **Table S1**. Differentially expressed genes involved in angiogenesis, as identified by the MetaCore program. Fold change between *Klf5*-wildtype and *Klf5*-null groups are shown for each of the genes. P and R indicate promoting and repressive functions, respectively, of a gene in angiogenesis. | | | |
| --- | --- | --- | --- |
| **Gene symbol** | **RefSeq** | **Fold change** | **Function** |
| Erbb4 | NM_010154 | 3.88053 | P |
| Cited1 | NM_007709 | 3.31573 | P |
| Agtr1a | NM_177322 | 3.30762 | P |
| Egf | NM_010113 | 2.97239 | P |
| S1pr3 | NM_010101 | 2.48751 | P |
| Adora2b | NM_007413 | 2.30257 | P |
| Ednrb | NM_007904 | 2.15406 | P |
| Hey1 | NM_010423 | 2.00228 | P |
| Il1a | NM_010554 | 1.93414 | P |
| Vcam1 | NM_011693 | 1.82136 | P |
| Pdgfd | NM_027924 | 1.81535 | P |
| Pdgfrb | NM_001146268 | 1.80956 | P |
| Grb14 | NM_016719 | 1.7364 | P |
| Flt1 | NM_010228 | 1.73543 | P |
| Pde3a | NM_018779 | 1.73236 | P |
| Cxcr2 | NM_009909 | 1.71898 | P |
| Il1b | NM_008361 | 1.70608 | P |
| Pfkfb3 | NM_001177753 | 1.69234 | P |
| Angpt2 | NM_007426 | 1.66491 | P |
| Cp | NM_001042611 | 1.62802 | P |
| Pgf | NM_008827 | 1.61962 | P |
| Emcn | NM_001163522 | 1.59372 | P |
| Angptl4 | NM_020581 | 1.57521 | P |
| Pdgfb | NM_011057 | 1.57034 | P |
| Nrp1 | NM_008737 | 1.54758 | P |
| Tek | NM_013690 | 1.5265 | P |
| Pde2a | NM_001143848 | -1.51908 | P |
| Foxc1 | NM_008592 | -1.62876 | P |
| Npy2r | NM_008731 | -1.68248 | P |
| Klk1b24 | NM_010643 | -1.74021 | P |
| Chrm2 | NM_203491 | -1.75509 | P |
| L1cam | NM_008478 | -2.47281 | P |
| Tbx1 | NM_011532 | -2.63012 | P |
| Trf | NM_133977 | 2.22695 | R |
| Sstr2 | NM_009217 | 2.1022 | R |
| Ltf | NM_008522 | -5.80334 | R |
