# Supplementary figures and images for: KLF5 inhibits angiogenesis in PTEN-deficient prostate cancer by attenuating AKT activation and subsequent HIF1α accumulation
Source: Mol Cancer. 2015 Apr 21;14:91. doi: 10.1186/s12943-015-0365-6 (PMC4417294; doi:10.1186/s12943-015-0365-6)

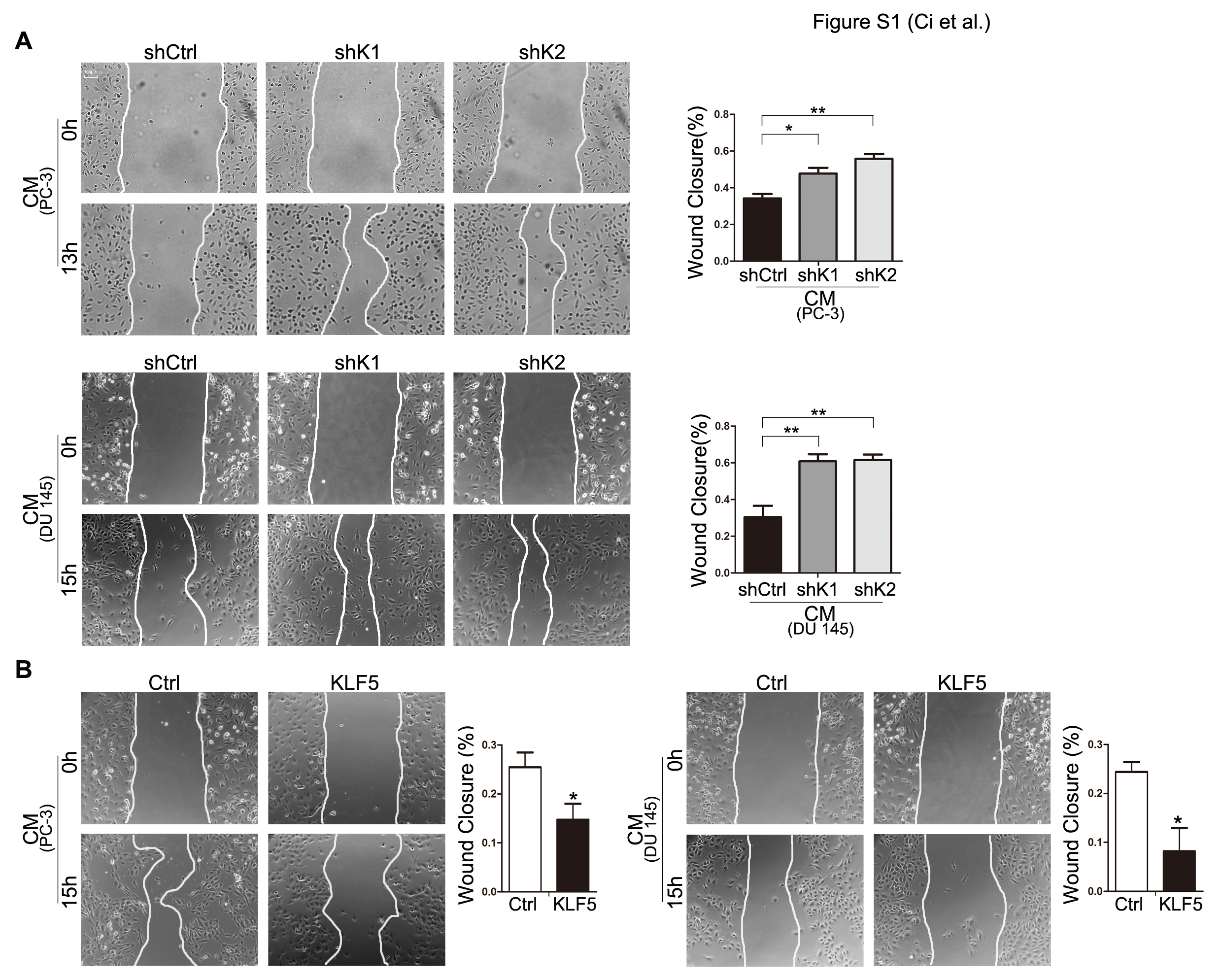

Supplement: Additional file 2: Figure S1. — Expression changes in KLF5 in PC-3 and DU 145 prostate cancer cells modulate wound healing in HuVECs. Confluent HuVECs cultured in conditioned media were scratched with a pipette tip and photographed from 4 fields at 0 and 13-15 hours for each sample. shK1 and shK2 indicate two shRNAs targeting KLF5 (sh36 and sh37) (A), Ctrl and KLF5 indicate lentiviruses expressing the pSin vector control and KLF5, respectively (B). Areas of wound were measured by using ImageJ software to indicate the extent of wound closure. *, P<0.05; **, P<0.01. [file 12943_2015_365_MOESM2_ESM.tiff]

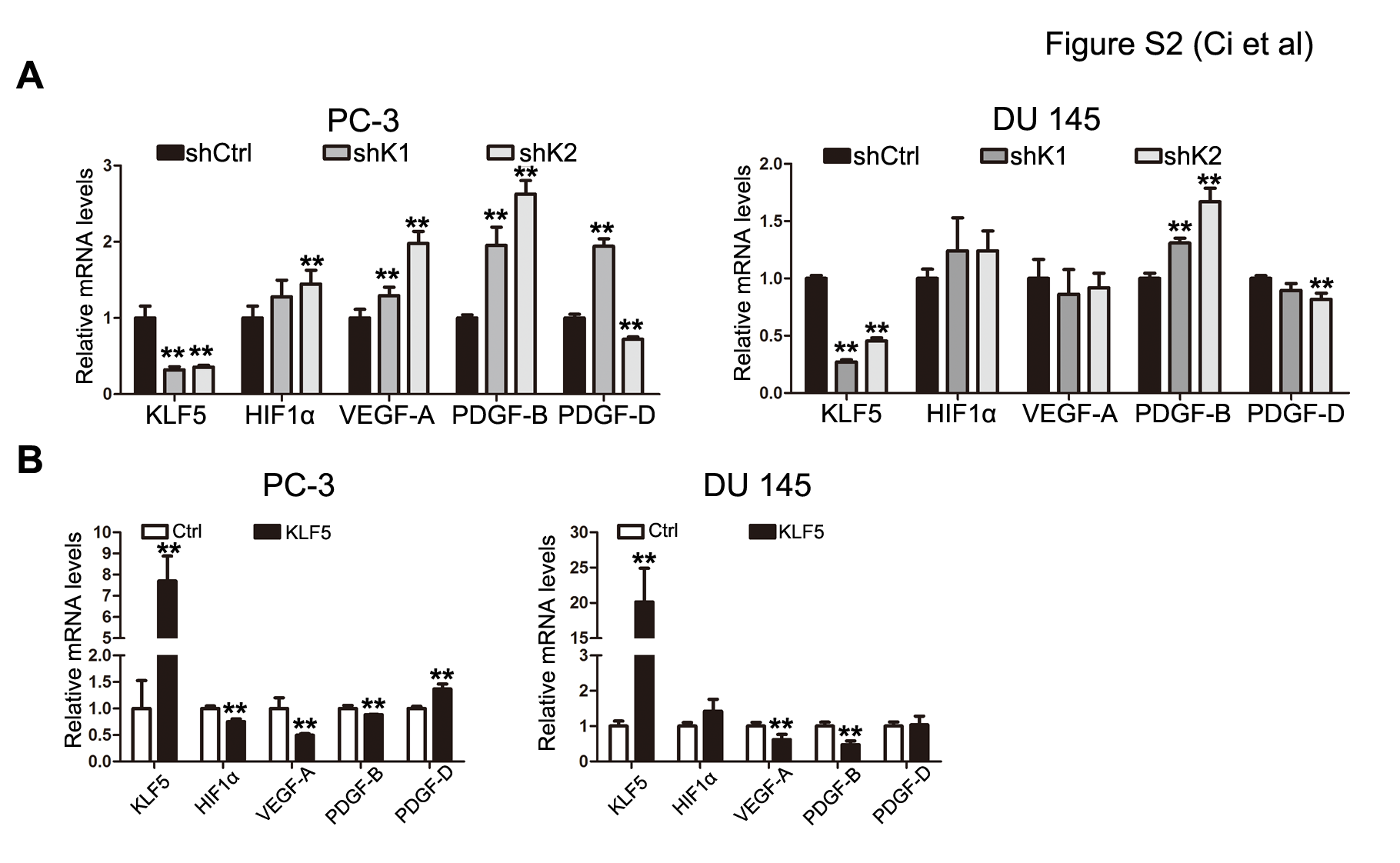

Supplement: Additional file 3: Figure S2. — Modulation of KLF5 expression affects mRNA expression of pro-angiogenic factors. Real-time RT-PCR was used to detect mRNA expression of HIF1α, VEGF-A, PDGF-B and PDGF-D mRNA in PC-3 and DU 145 prostate cancer cells with stable knockdown (A) or ectopic expression (B) of KLF5. shK1 and shK2 indicate two shRNAs targeting KLF5 (sh36 and sh37) (A), Ctrl and KLF5 indicate pSin vector control and pSin-KLF5 (B), respectively. *, P<0.05; **, P<0.01. (TIFF 334 kb) [file 12943_2015_365_MOESM3_ESM.tiff]

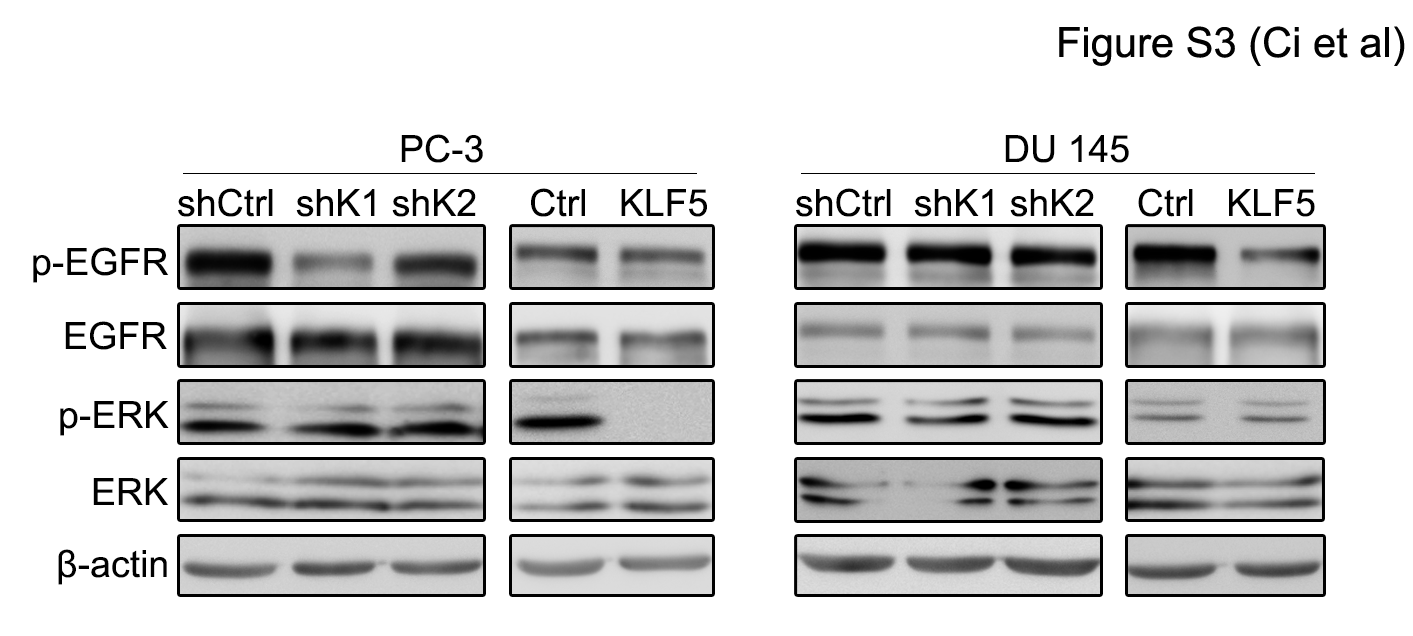

Supplement: Additional file 4: Figure S3. — EGFR and ERK activation do not appear to mediate the upregulation of HIF1α by KLF5 knockdown in human prostate cancer cell lines. Protein expression of p-EGFR (Y1068), EGFR, p-ERK and ERK were determined by Western blotting in PC-3 and DU 145 cells with stable knockdown or ectopic expression of KLF5. shK1 and shK2 indicate two shRNAs targeting KLF5 (sh36 and sh37), and Ctrl and KLF5 indicate the pSin vector control and pSin-KLF5 expression construct, respectively. [file 12943_2015_365_MOESM4_ESM.tiff]
