# Supplementary material for: KLF5 inhibits angiogenesis in PTEN-deficient prostate cancer by attenuating AKT activation and subsequent HIF1α accumulation
Source: Mol Cancer. 2015 Apr 21;14:91. doi: 10.1186/s12943-015-0365-6 (PMC4417294; doi:10.1186/s12943-015-0365-6)
Supplement: Additional file 5: Table S2. — Primer sequences used in this study. [file 12943_2015_365_MOESM5_ESM.docx]

| **Table S2**. Primer sequences used in this study. | |  |
| --- | --- | --- |
| Gene name | Forward primer/reverse primer (PCR product size in bp) |  |
| Hif1α | 5'-TCAAGTCAGCAACGTGGAAG-3'/5'-TATCGAGGCTGTGTCGACTG-3' (198) |  |
| Vegf-a | 5'-AAGGAGAGCAGAAGTCCCATGA-3'/5'-CTCAATCGGACGGCAGTAGCT-3' (74) |  |
| Vegfr1 | 5'-GAGGAGGATGAGGGTGTCTATAGGT-3'/5'-GTGATCAGCTCCAGGTTTGACTT-3' (116) |  |
| Vegfr2 | 5'-ACAGACCCGGCCAAACAA-3'/5'-TTCCCCCCTGGAAATCCTC-3' (69) |  |
| Pdgf-b | 5'-TGTTCCAGATCTCTCGGAAC-3'/5'-GCGGCCACACCAGGAAG-3' (60) |  |
| Pdgf-d | 5'-CAATTCGGACTAGAGGAAGCAG-3'/5'-CTTCCGGTTGGAAATCTTCCAC-3' (238) |  |
| Pdgfrb | 5'-GTGGTGAACTTCCAATGGACG-3'/5'-GTCTGTCACTGGCTCCACCAG-3' (66) |  |
| Gapdh | 5'-CCAGCCTCGTCCCGTAGACA-3'/5'-GCCGTTGAATTTGCCGTGAG-3' (190) |  |
| HIF1α* | 5'-GAAAGCGCAAGTCCTCAAAG-3'/5'-TGGGTAGGAGATGGAGATGC-3' (167) |  |
| VEGF-A* | 5'-CTACCTCCACCATGCCAAGT-3'/5'-GCAGTAGCTGCGCTGATAGA-3' (109) |  |
| PDGF-B* | 5'-AATGGTCACCCGAGTTTGG-3'/5'-CTGGCATGCAAGTGTGAGAC-3' (105) |  |
| PDGF-D* | 5'-GGATGGTCTCATCTCTTCGG-3'/5'-CAGAGCGCATCCATCAAAG-3' (94) |  |
| GAPDH* | 5'-GTGGTCCAGGGGTCTTACTC-3'/5'-TTCAACAGCGACACCCACTC-3' (167) |  |
| KLF5* | 5'-AAGGAGTAACCCCGATTTGG-3'/5'-CAGCCTTCCCAGGTACACTT-3' (147) |  |
| Note: * indicates a human gene. | |  |

­­
